# Detecting eukaryotic microbiota with single-cell sensitivity in human tissue

**DOI:** 10.1186/s40168-018-0529-x

**Published:** 2018-09-01

**Authors:** Susanne Lager, Marcus C. de Goffau, Ulla Sovio, Sharon J. Peacock, Julian Parkhill, D. Stephen Charnock-Jones, Gordon C. S. Smith

**Affiliations:** 1 Department of Obstetrics and Gynaecology, University of Cambridge, National Institute for Health Research Cambridge Biomedical Research Centre, Cambridge, UK; 2 Centre for Trophoblast Research (CTR), Department of Physiology, Development and Neuroscience, University of Cambridge, Cambridge, UK; 3 Wellcome Trust Sanger Institute, Cambridge, UK; 4 Department of Medicine, University of Cambridge, Cambridge, UK; 5 London School of Hygiene and Tropical Medicine, London, UK

**Keywords:** 18S rRNA gene, Sequencing, Infection, Placenta, Pregnancy complication, Fetal growth restriction, Pre-eclampsia, Pre-term birth

## Abstract

**Background:**

Fetal growth restriction, pre-eclampsia, and pre-term birth are major adverse pregnancy outcomes. These complications are considerable contributors to fetal/maternal morbidity and mortality worldwide. A significant proportion of these cases are thought to be due to dysfunction of the placenta. However, the underlying mechanisms of placental dysfunction are unclear. The aim of the present study was to investigate whether adverse pregnancy outcomes are associated with evidence of placental eukaryotic infection.

**Results:**

We modified the 18S Illumina Amplicon Protocol of the Earth Microbiome Project and made it capable of detecting just a single spiked-in genome copy of *Plasmodium falciparum*, *Saccharomyces cerevisiae*, or *Toxoplasma gondii* among more than 70,000 human cells. Using this method, we were unable to detect eukaryotic pathogens in placental biopsies in instances of adverse pregnancy outcome (*n* = 199) or in healthy controls (*n* = 99).

**Conclusions:**

Eukaryotic infection of the placenta is not an underlying cause of the aforementioned pregnancy complications. Possible clinical applications for this non-targeted, yet extremely sensitive, eukaryotic screening method are manifest.

**Electronic supplementary material:**

The online version of this article (10.1186/s40168-018-0529-x) contains supplementary material, which is available to authorized users.

## Background

Fetal growth restriction (FGR), pre-eclampsia, and pre-term birth are major adverse pregnancy outcomes, and a significant proportion of cases are thought to be due to placental dysfunction. However, the mechanisms underlying this placental dysfunction remain obscure. Infections during pregnancy may result in adverse outcomes. Pregnancy is associated with increased susceptibility to certain infections, including candidiasis and *Plasmodium falciparum* [1–3]. Furthermore, protozoal infections are a significant contributing factor in stillbirths [4]. *Plasmodium falciparum* infection has also been associated with maternal death, reduced fetal growth, and pre-term births. Generally, pregnancy outcomes are poorer with increasing placental *P*. *falciparum* infection [4]. As part of a systematic study using culture-free methods to determine whether these disorders may be caused by microbial or viral pathogens, we set out to investigate whether adverse pregnancy outcome was associated with evidence of placental eukaryotic infection.

One molecular method to detect eukaryotic pathogens is amplification and sequencing of 18S rRNA genes. When we addressed this aim, we initially employed the methodological approach described by the Earth Microbiome Project (EMP) [5, 6]. However, the context of EMP analyses frequently involves niches where the microbial eukaryotic biomass is abundant compared to other 18S rRNA signals. Detection of 18S rRNA genes from potential pathogens in human biopsies is problematic as (i) even low levels of signal would be highly relevant and (ii) the 18S rRNA gene is also present in the human genome (unlike the bacterial 16S rRNA gene). Consequently, development and application of PCR-based diagnostic methods for human eukaryotic infections has been slow [7, 8], resulting in a dearth of well-conducted 18S rRNA gene sequencing studies, in contrast to the numerous 16S rRNA gene sequencing studies. Shotgun-metagenomics analyses similarly suffer from the fact that close to 100% of the DNA obtained from human biopsies is human, greatly reducing the detection sensitivity of (rare) eukaryotic pathogens. Hence, we first set out to develop a modified approach for the 18S Illumina Amplicon Protocol of the EMP which would maximize the signal from potential eukaryotic pathogens over the human host. Second, we employed the optimized protocol in order to determine whether pregnancies complicated by FGR, pre-eclampsia, or pre-term birth are associated with eukaryotic infection of the placenta.

## Methods

### Ethics

The Pregnancy Outcome Prediction study (POPs) was approved by the Cambridgeshire 2 Research Ethics Committee (reference number 07/H0308/163). All study participants gave informed written consent. The design and conduct of the study are described in detail elsewhere [9]. The characteristics of the eligible women and those who participated in POPs have also previously been described [10].

### Patient selection

From the POPs cohort [9, 11], placentas from 60 healthy, term deliveries (≥ 37 weeks’ gestation) were selected for a pilot study to investigate the influence of mode of delivery and sample collection time on eukaryotic microbes detected (clinical characteristics are presented in Additional file 1: Table S1).

To study associations between placental eukaryotic infection and pregnancy complications, cases of FGR (less than third percentile based on customized birth weight accounting for gestational age, fetal sex, maternal weight/height, ethnicity, parity, and smoking [12] (*n* = 50) or pre-eclampsia according to the 2013 ACOG Guidelines (The American Congress of Obstetricians and Gynecologists [13]; *n* = 49)) were selected (Additional file 1: Table S2). All pre-eclampsia cases displayed severe features of pre-eclampsia; specifically, they had either severe hypertension (systolic blood pressure ≥ 160 mmHg, diastolic blood pressure ≥ 110 mmHg) or evidence of hepatic, renal, hematologic, cerebral, or pulmonary complications [13]. Cases were matched one-to-one with healthy controls (*n* = 99). Cases and controls were all term deliveries (≥ 37 weeks’ gestation). The matching criteria were the following: mode of delivery (absolute match) and as close as possible for maternal BMI, maternal age, gestational age, sample collection time (i.e., the interval between birth and collection of the placenta), maternal smoking, and fetal sex. In addition, placentas from 100 pre-term deliveries (< 37 weeks’ gestation) were selected. Clinical characteristics are presented in Additional file 1: Table S2.

### Placenta collection

Placental villous tissue was collected after delivery as previously described in detail [9]. Briefly, after removal of the basal plate, villous tissue was obtained from four different lobules of the placenta. The selected tissue samples had no visible damage, hematomas, or infarctions. Maternal blood was removed by washing the tissue samples in ice-cold phosphate-buffered saline. Tissue was rapidly frozen in liquid nitrogen and stored at − 80 °C until further processing. For the DNA isolation, approximately 25 mg of villous tissue was cut from the stored tissue. In the pilot study, tissue from the four biopsy collection points were studied combined (*n* = 60), as well as separate biopsies from four of these women (*n* = 44; 11 biopsies each from four placentas). In the pregnancy complication study, tissue from all four biopsy collection points were combined (*n* = 298). To reduce the risk of environmental contamination of the samples, the processing was performed in a Class 2 biological safety cabinet with single-use sterile forceps and scalpels. Each matched case-control pair was processed in parallel together on the same day. For all subsequent steps, the case-control pairs were processed in parallel using the same lot of reagents.

### DNA isolation

DNA was isolated from placental tissue with the Fast DNA Spin kit (MP Biomedical, Santa Ana, CA, USA). Tissue was homogenized by bead-beating (2 cycles of 40 s, speed 6.5 on a FastPrep-24; MP Biomedical). Samples were placed on ice for 5 min between each bead-beating cycle. To protect the samples from possible environmental contamination, the DNA isolation was also performed in a Class 2 biological safety cabinet. Cabinet and pipettes were cleaned with DNA AWAY Surface Decontaminant (Fisher Scientific, Loughborough, UK). Gloves changed between handling each sample. All plastics used for the DNA extraction, as well as subsequent steps, not supplied in the kits were nuclease-free: PCR clean 2.0 and 1.5 ml DNA LoBind Tubes (Eppendorf, Hamburg, Germany) and nuclease-free filter tips (TipONE sterile filter tips, STARLAB (UK), Ltd., Milton Keynes, UK). Extraction blanks were performed for each box of DNA isolation kit used. These DNA extraction blanks, or negative controls, did not contain any added biological material, solely the reagents of the DNA isolation kit subjected to the complete DNA extraction procedure (bead-beating, matrix binding, spin filtering, washing, and elution of nucleic acids). The DNA extraction blanks were subjected to the entire analysis protocol alongside the placental samples: DNA isolation, 18S rRNA gene PCR amplification, sequencing, and data analysis. Matched case-control pairs were processed together on the same day, using the same DNA isolation kit batch. DNA concentrations were determined by Nanodrop Lite (ThermoFisher Scientific, Waltham, MA, USA).

### Positive control experiments

Isolated DNA from *Plasmodium falciparum* (kind gift from Dr. Julian Rayner, Wellcome Trust Sanger Institute), *Toxoplasma gondii* (cat# 50174D; ATCC, Manassas, VA, USA), and *Saccharomyces cerevisiae* (strain Y7092; kind gift from Dr. Nianshu Zhang, University of Cambridge) was added in a randomized order (1, 10, 100, 1000, or 10,000 genome copies) per 500 ng of DNA isolated from human placenta (equivalent to > 70,000 cells). A total of approximately 500 ng DNA was used for each of the detection limit assays (18S rRNA gene sequencing and HiSeq X Ten sequencing).

### EMP protocol PCR amplification of 18S rRNA gene

The V9 region of the 18S rRNA gene was amplified with primers: Forward-1391 5′- AAT GAT ACG GCG ACC ACC GAG ATC TAC ACT ATC GCC GTT CGG TAC ACA CCG CCC GTC-3′ and Reverse-EukBr 5′- CAA GCA GAA GAC GGC ATA CGA GAT *nnn nnn nnn nnn* AGT CAG TCA GCA TGA TCC TTC TGC AGG TTC ACC TAC-3′ [the *n* string represents a unique 12-mer barcode]. In order to reduce amplification of human 18S rRNA gene, a blocking primer was included during the PCR reaction (5′-GCC CGT CGC TAC TAC CGA TTG G*II III* TTA GTG AGG CCC T-3SpC3; each *I* indicates deoxyInosine, and “SpC3” a C3 Spacer phosphoramidite at the 3′-end of the primer which prevents primer extension). The 18S rRNA gene amplifications were carried out with 5 Prime HotMasterMix (5 Prime GmbH, Hamburg, Germany). The Primer design and the PCR amplification protocol have previously been described by the EMP (http://www.earthmicrobiome.org/) and used in publications [14–16].

### Q-PCR optimization of 18S rRNA gene PCR conditions

Known amounts of DNA from *S*. *cerevisiae* or *P*. *falciparum* were added to human DNA isolated from placenta. Quantification of 18S PCR amplicons was carried out in triplicate on a 7900HT Fast Real-Time PCR System (Applied Biosystems, Foster City, CA, USA). The 18S rRNA amplicons were detected with human forward 5′-CTA CTA CCG ATT GGA TGG TT-3′ (final conc. 0.5 μM), human reverse 5′-TCA AGT TCG ACC GTC TTC-3′ (final conc. 0.5 μM), human probe [6FAM]-TAG TGA GGC CCT CGG ATC GGC-[MGB/NFQ] (final conc. 0.25 μM), *S*. *cerevisiae* forward 5′-GTC GCT AGT ACC GAT TGA A-3′ (final conc. 0.1 μM), *S*. *cerevisiae* reverse 5′-TCC AAA TTC TCC GCT CTG-3′ (final conc. 0.1 μM), and *S*. *cerevisiae* probe [6FAM]-AGC AGA TCC TGA GGC CTC ACT AAG C-[MGB/NFQ] (final conc. 0.1 μM). The PCR cycling conditions to detect human 18S PCR amplicons were an initial step of 95 °C for 10 min followed by 30 cycles of 95 °C (15 s) and 63 °C (1 min). The PCR to detect *S*. *cerevisiae* 18S PCR amplicons was run with an initial step of 95 °C for 10 min followed by 30 cycles of 95 °C (15 s) and 60 °C (1 min). TaqMan Universal master mix (Life Technologies, Carlsbad, CA, USA) was used in the amplifications.

### Optimized dual-index approach for 18S rRNA gene amplification and sequencing

The 18S rRNA gene was amplified in triplicate (pilot experiment) or quadruplicate (pregnancy complication cohort) reactions with 180 ng DNA/reaction. The final concentration of the forward and reverse primers was 0.2 μM and of the blocking primer 3.2 μM. All primers were purchased from Eurofins Genomics (Ebersberg, Germany). Working aliquots of the primers were prepared in a Class 2 biological safety cabinet (cleaned with DNA AWAY Surface Decontaminant), in PCR clean nuclease-free DNA LoBind Tubes (Eppendorf) with nuclease-free filter tips (TipONE sterile filter tips, STARLAB) and Tris-EDTA buffer (Sigma-Aldrich Company Ltd., Gillingham, UK). In the single-index approach, a barcode was present only on the reverse primer. In the dual-index approach, a barcode was included in the forward primer as well: Barcoded forward-1391: 5′-AAT GAT ACG GCG ACC ACC GAG ATC TAC CA*n nnn nnn nnn nn*T ATC GCC GTT CGG TAC ACA CCG CCC GTC-3′ [the *n* string represents unique 12-mer barcode]. 18S rRNA gene amplifications were carried out with 5 Prime HotMasterMix (5 Prime GmbH) and UltraPure DNase/RNase-Free Water (Thermo Fisher Scientific) in 0.2 ml PCR strips (STARLAB). PCR amplification was carried out on a SureCycler 8800 Thermal Cycler (Agilent Technologies, Stockport, UK). PCR amplification comprised an initial step of 94 °C for 3 min followed by 30 cycles of 94 °C (45 s), 65 °C (15 s), 57 °C (30 s), and 72 °C (90 s). After completion of cycling, the reactions were incubated for 10 min at 72 °C. After completion of the PCR, replicates of each sample were pooled, with DNA precipitated in a mixture of 1 M NaCl and 99.5% ethanol (Sigma-Aldrich) overnight. Samples were spun at 14,000*g* for 20 min and the pellet washed in 70% ethanol and spun at 14,000*g* for 12 min. The pellet was air-dried in a Class 2 biological safety cabinet before being resuspended in Tris-EDTA buffer (Sigma-Aldrich) overnight. DNA concentrations were determined by Qubit fluorometry (Invitrogen, Carlsbad, CA, USA). Equimolar pools of the PCR amplicons were run on 1% agarose/TBE gels, DNA was visualized with ethidium bromide, and excised bands were cleaned up with a Wizard SV Gel and PCR Clean-Up System (Promega UK, Southampton, UK). The equimolar pools were sequenced on the Illumina MiSeq platform using paired-end 250 cycle MiSeq Reagent Kit V2 (Illumina, San Diego, CA, USA).

### 18S MiSeq data analysis

Quality control and merging of paired reads was performed with PEAR (v0.9.5) using its default settings [17] except for a minimum overlap size of 40 base pairs. Due to the limited size of the V9 region, there is a full overlap of the entire sequenced region. Merged reads were subsequently analyzed using the QIIME assign_taxonomy.py script (v1.9.1) using the mothur assignment method option and the 18S Silva_119_rep_set97_18S.fna reference library [18–20]. The first MiSeq run, which used only a single-index on the reverse primer, revealed that *P*. *falciparum* and *T*. *gondii* reads, likely originating from the positive control samples, were detected in placental samples that should not have any of these reads. To verify the origins of these incorrectly assigned reads and the likely mechanism of the incorrect read assignment, a dual-index sequencing approach was instead implemented on these same samples. This repeat analysis using a dual index was also analyzed by the Illumina MiSeq platform as if there was still only a single index on the reverse primer, using the same CSV input file, to see if the previous results could be replicated. The index on the forward primer could then subsequently be utilized to see where these errant reads originated from, due to the limited size of the V9 region. In this analysis, matching or non-matching forward barcodes were searched for within the 250 nucleotide long reverse reads by searching nucleotide positions 110–155 for the presence of the correct forward barcode using the Excel function IF (ISNUMBER (SEARCH(“barcode”,“read”)),true,false). Both “false” and “true” reads were separated and put into individual reverse read fastq files, matched with their respective forward reads using the makepairs function of git.io/pairfq_lite and were subsequently analyzed as described above with PEAR and QIIME. Merged “false” reads which were identified as either *P*. *falciparum* or *T*. *gondii* were double-checked to verify that the index on their forward primer indeed corresponded to one of the forward indexes used for the positive controls by again searching nucleotide positions 110–155 of the corresponding (unmerged) reverse read for a barcode used in one of the positive controls. A dual-index approach was used for all subsequent MiSeq runs providing the Illumina MiSeq platform with a dual-index CSV file.

### HiSeq X Ten data analysis

The same positive control samples used for the MiSeq 18S experiments were also analyzed by whole genome sequencing using the Illumina HiSeq X Ten. For the library preparation of the HiSeq X Ten platform sample, DNA levels were first quantified with a Biotium Accuclear Ultra High Sensitivity dsDNA Quantitative kit (Cambridge Bioscience, Bar Hill, Cambridge, UK) using the Mosquito LV liquid platform (TTP Labtech, Melbourn, Cambridgeshire, UK), Bravo WS (Agilent, Santa Clara, CA, USA), and BMG FLUOstar Omega plate reader (BMG Labtech, Aylesbury, Buckinghamshire, UK) and cherrypicked to 200 ng/120 μl using a Tecan liquid handling platform (Tecan, Männedorf, Switzerland). Cherrypicked plates were sheared to 450 bp using a Covaris LE220 instrument (Covaris, Woburn, Massachusetts, USA). Post-sheared samples were purified using Agencourt AMPure XP SPRI beads (Beckman Coulter, Brea, CA, USA) on an Agilent Bravo WS (Agilent). Library construction (ER, A-tailing and ligation) was carried out using a “NEB Ultra II custom kit” (NEB, Ipswich, Massachusetts, USA) on an Agilent Bravo automation system (Agilent). The PCR was carried out with KapaHiFi Hot start mix (Kapa Biosystems, Wilmington, Massachusetts, USA) and IDT 96 iPCR tag barcodes (Integrated DNA Technologies, Coralville, Iowa, USA) using the Agilent Bravo WS automation system (Agilent). The PCR amplification profile was an initial step of 95 °C for 5 min followed by 5 cycles of an initial step of 98 °C (30 s), 30 s at 65 °C, and 1 min at 72 °C. After completion of cycling, the reactions were incubated for 10 min at 72 °C. DNA from the post-PCR plate was purified using Agencourt AMPure XP SPRI beads (Beckman Coulter) on a Beckman BioMek NX96 liquid handling platform (Beckman Coulter). Libraries were again quantified with the Biotium Accuclear Ultra High Sensitivity dsDNA Quantitative kit (Cambridge Bioscience) using the Mosquito LV liquid platform (TTP Labtech), Bravo WS (Agilent), and BMG FLUOstar Omega plate reader (BMG Labtech). Libraries were pooled in equimolar amounts using a Beckman BioMek NX-8 liquid handling platform (Beckman Coulter) and were normalized to 2.8 nM for cluster generation on a c-BOT and loading onto the Illumina HiSeq X Ten platform (Illumina). Sequencing of the samples (150 bp, paired end) resulted in 402 million pairs of reads on average per sample (range 388–420 million, 3.2 billion total paired reads in lane). On average, 97.6% of all reads (range 97–98%) were initially mapped to the host sequence (human reference genome) with Bowtie 2 (v2.2.3, default settings) and were subsequently removed. A custom Kraken (v0.10.6) [21] reference database was built in order to detect any potential non-human eukaryotic signal using metagm_build_kraken_db and -max_db_size 30. This custom Kraken reference database included both the default bacterial and viral libraries, and an accessions.txt file was supplied (via -ids_file) containing a diverse array of organisms chosen from all sequenced forms of eukaryotic life (see Additional file 1: Table S3 for accession numbers). This wide array was chosen to both detect potentially relevant unknown organisms and to identify human reads which had not been mapped to the human reference genome. Kraken was run using the metagm_run_kraken option. Apparent non-human eukaryotic signals were found in every placental sample at a similar percentage, and on further analysis, these turned out to be human reads that were not removed when mapped against the human reference genome; this was apparent as Kraken mapped most of these reads to the various Animalia (Chordata) species (see Additional file 1: Table S3). Additional in-depth assembly-based taxonomic analyses would have been performed if an unexpected relevant non-human eukaryotic signal was detected, but none were found.

### Statistics and positive control data normalization

Differences between groups when optimizing the 18S rRNA PCR conditions were evaluated by repeated measures ANOVA. *P* values less than 0.05 were considered statistically significant. Statistical calculations were performed in GraphPad Prism 7 (GraphPad Software, Inc., La Jolla, CA, USA).

The signals of the various eukaryotic signatures in the positive control samples were normalized based upon the remaining proportion of human 18S reads. This normalization utilizes the fact that all samples contain the same amount of human DNA (same weight of tissue per sample). However, quantifying reads relative to the human signal required correction for the size of any non-human signal. This is required as large non-human signals significantly lower the percentage of human reads detected. To illustrate, if a sample only contains human DNA, approximately 99.9% of all reads will be taxonomically assigned as being from a human after sequencing. If a sample also contains, for example, a large *S*. *cerevisiae* signal, accounting for ~ 50% of all reads, this will depress all other smaller positive control signals by a factor of ~ 2. To account for signal depression of and due to the three eukaryotic spiked species in the various positive control samples, each positive control sample was first normalized to an arbitrary number of 100,000 reads in order to compare them in subsequent steps. Secondly, a correction factor (CF) is calculated based upon the total eukaryotic signal strength in a positive control or in other words on the depression of the percentage of human reads (which represent a fixed eukaryotic signal strength in each sample) by other eukaryotic signals as follows:

$$ \mathrm{Correction}\ \mathrm{factor}=\frac{1+\left(99.9-\mathrm{Human}\ \left(\%\right)\right)}{\mathrm{Human}\ \left(\%\right)} $$

The 99.9% refers to the percentage of reads that would be taxonomically assigned as being from a human in a placental sample without any microbial signature. The normalized number of reads for each eukaryote-detected signal is multiplied by the correction factor (step 2) to calculate a value for the signal strength of each eukaryotic signal (*P*. *falciparum*, *S*. *cerevisiae*, and *T. gondii*) that can be directly compared with the corresponding eukaryotic signals from the other positive controls. For example, if *S*. *cerevisiae* accounts for 50% of all reads, *P*. *falciparum* accounts for 20% of all the reads, and *T. gondii* accounts for 5% of all reads, and where the remainder is human, the correction factor would be ~ 4, and hence, the adjusted output would be approximately 50,000 × 4 = 200,000 for *S*. *cerevisiae*, 20,000 × 4 = 80,000 for *P*. *falciparum*, 5000 × 4 = 20,000 for *T. gondii*, and 100,000 for *Homo sapiens*.

## Results

### Optimization of 18S PCR conditions

**Fig. 1:**
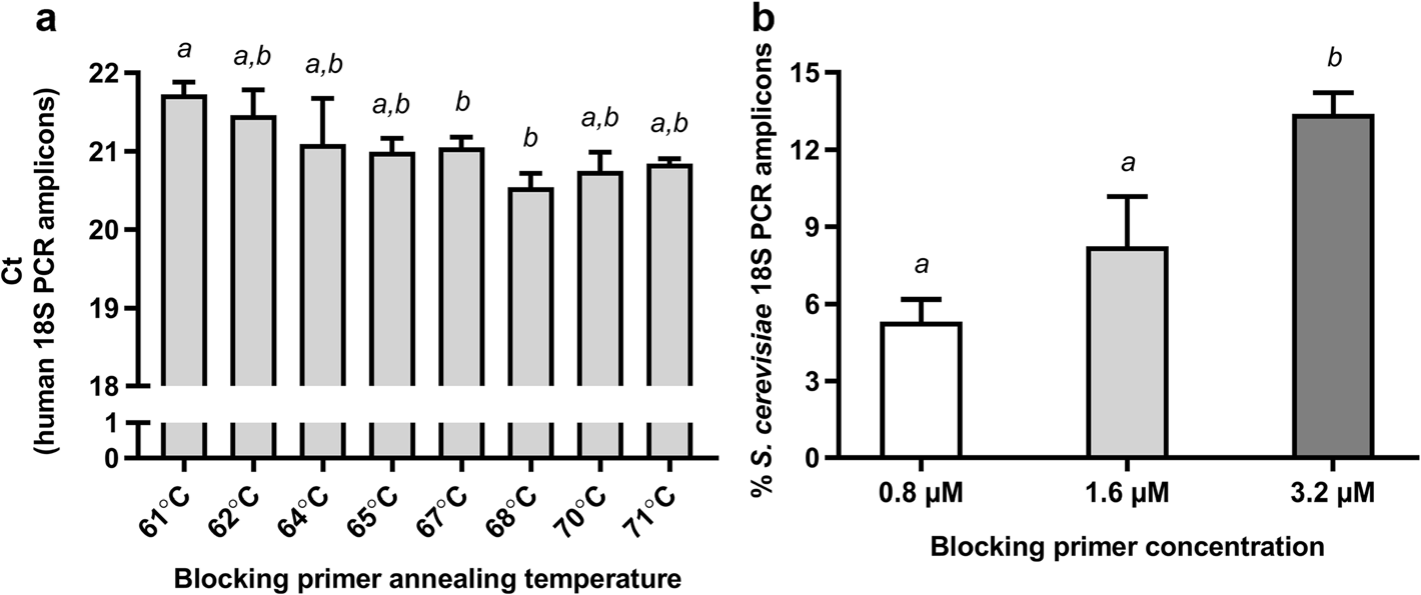
Optimization of blocking primer annealing temperature and concentration. The EMP protocol utilizes a mammal blocking primer annealing temperature of 65 °C and blocking primer concentration of 1.6 μM. **a** Different blocking primer annealing temperatures were tested for the 18S rRNA gene amplification. In all reactions, 1.6 μM blocking primer was used. The effect of increasing annealing temperature was determined by quantifying the yield of product by Q-PCR. A higher Ct indicates more effective blocking of amplification of the human 18S rRNA gene. An annealing temperature of 61 °C resulted in a significantly higher Ct than at 67 °C or 68 °C (repeated measures ANOVA followed by Tukey’s multiple comparisons test). Data presented as mean + SEM, *n* = 3; different letters above bars indicate *P* < 0.05. **b** Comparison of blocking primer concentration and annealing temperature (65 °C) with the original sample of *S*. *cerevisiae* of 0.0001%. Data presented as mean + SEM, *n* = 4. Different letters above the bars indicate *P* < 0.05 (repeated measures ANOVA followed by Tukey’s multiple comparisons test)

To increase the sensitivity of the EMP protocol in human tissue samples, mammal blocking primer concentrations and annealing temperature were first optimized to maximize the ratio of non-human to human 18S reads. The optimization was performed using a range of *P*. *falciparum* or *S*. *cerevisiae* DNA concentrations (≤ 1%) added to human placental DNA samples. When comparing the samples with the lowest proportion of *S*. *cerevisiae*, doubling the blocking primer amount (3.2 μM) while keeping the EMP protocol’s annealing temperature (65 °C) resulted in optimal inhibition of human 18S rRNA gene amplification (Fig. 1 and Additional file 1: Figure S1).

### Single-index 18S rRNA sequencing

**Fig. 2:**
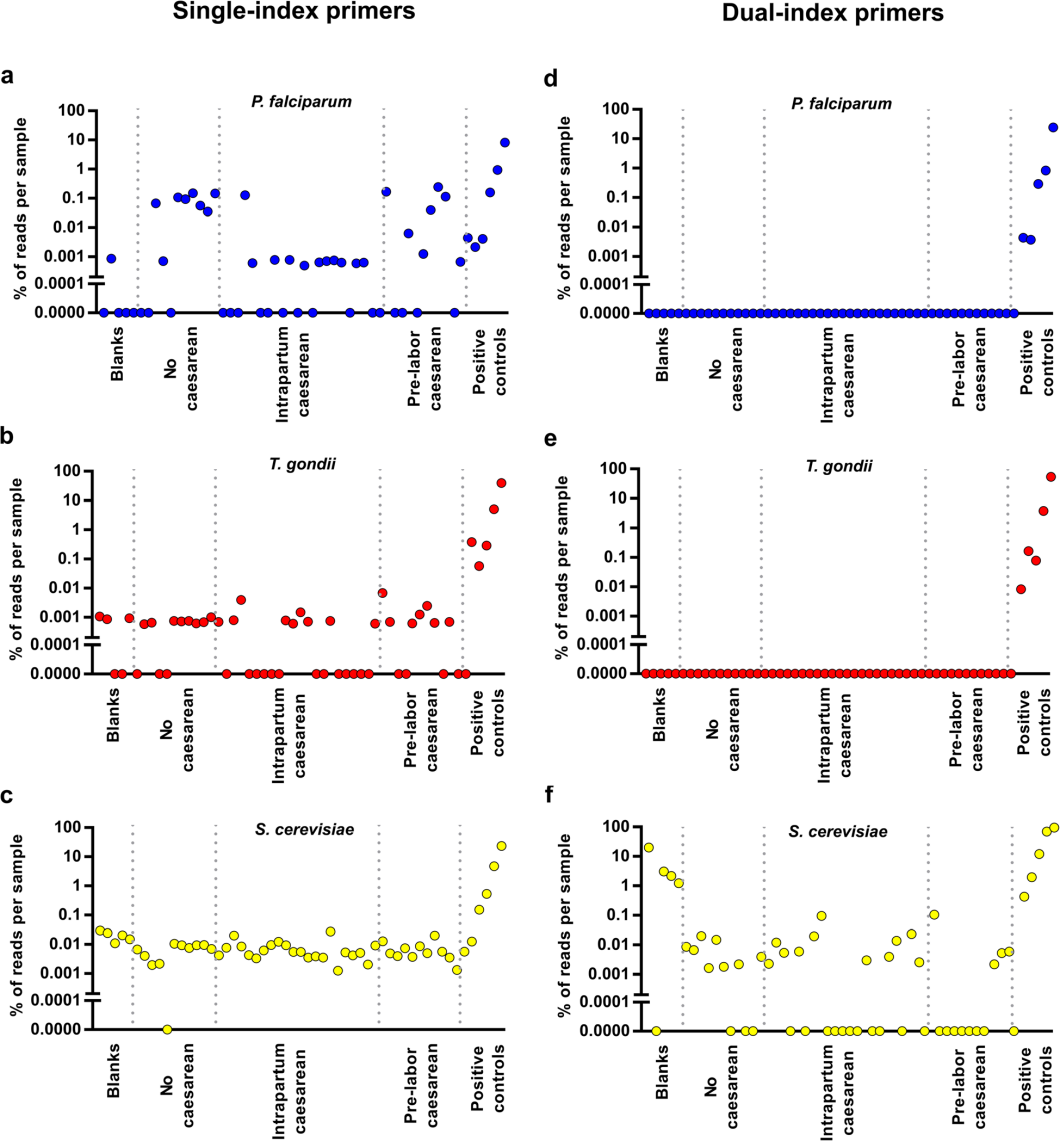
Detection of positive controls with single-index primers and dual-index primers. Detection of **a** *P*. *falciparum*, **b** *T*. *gondii*, and **c** *Saccharomyces (cerevisiae)* in healthy placental samples and positive controls with single-index primers. The same samples were re-analyzed with dual-index primers for detection of **d** *P*. *falciparum*, **e** *T*. *gondii*, and **f** *Saccharomyces (cerevisiae)*. Graphs illustrate kit blanks (left; *n* = 5), healthy placental samples (middle; *n* = 44 placental biopsies from four women), and positive controls (right [0 to 10,000 added genome copies ordered from left to right]; *n* = 6). With a dual-index sequencing approach, the *P*. *falciparum* and *T*. *gondii* signals disappear from all the healthy placental samples (*n* = 44) and the 0 genome copy control. Signal remains in the positive control samples (right side of graphs; *n* = 5). The high proportion of *S*. *cerevisiae* reads in blanks (**f**) was due to an overall low number of total reads in the blanks using dual-index primers. The absolute number of *Saccharomyces* 18S reads with the dual-index primers is presented in Additional file 1: Figure S3

Next, we studied three groups: (1) 44 placental samples obtained from four placentas delivered following a normal pregnancy (11 biopsies per placenta), (2) positive controls (placental samples with added *P*. *falciparum*, *T*. *gondii*, and *S*. *cerevisiae* DNA; *n* = 6), and (3) negative controls (blanks; Fig. 2a–c). Each of the samples was identified using single-index barcoded 18S rRNA amplicons, as per the EMP protocol, and all samples were pooled and sequenced in a single lane. We detected the pathogen signals in all of the positive controls. However, additionally, *P*. *falciparum* reads were detected in 27 out of the 49 other samples, including several strong signals (< 0.1% of reads) equivalent to 100-genome copies (Fig. 2a). Similarly, *T*. *gondii* reads were found in low amounts (~ 0.001% of reads) in 27 out of 49 samples (Fig. 2b). Finally, *Saccharomyces* reads were found to represent around 0.01% of all reads in 48 out of 49 samples, including all negative controls (Fig. 2c). It was apparent that we were obtaining false-positive results as (i) none of the four women providing healthy placental samples was documented as having any infection, (ii) multiple biopsies from the same woman were not consistently positive or negative for the given pathogen, (iii) the negative controls also demonstrated the presence of pathogen DNA, and (iv) we could not replicate the *P*. *falciparum* or *T*. *gondii* signals in a second sequencing run (Additional file 1: Figure S2). Notably, the second sequencing run did not include any positive control samples.

### Dual-index 18S rRNA sequencing

We hypothesized that the unexpected *P*. *falciparum* or *T*. *gondii* reads from the healthy placentas could be explained by primer cross-contamination and or mis-assignment, which leads to reads from one sample being incorrectly assigned to a different sample. Hence, we repeated the analysis of the same DNA samples but employed dual-index primer sequencing (i.e., a unique index at both ends of the amplicon). In contrast to the single-index primer approach, neither *P*. *falciparum* nor *T*. *gondii* reads were detected in placental samples except in the positive controls (Fig. 2d, e). All *P*. *falciparum* and *T*. *gondii* reads in placental samples for the single-index run originated from positive controls, due to index misattribution. In contrast, *Saccharomyces* reads were still detected in placental samples and negative controls (Fig. 2f and Additional file 1: Figure S3).

### Sensitivity of 18S rRNA and HiSeq X Ten sequencing

**Fig. 3:**
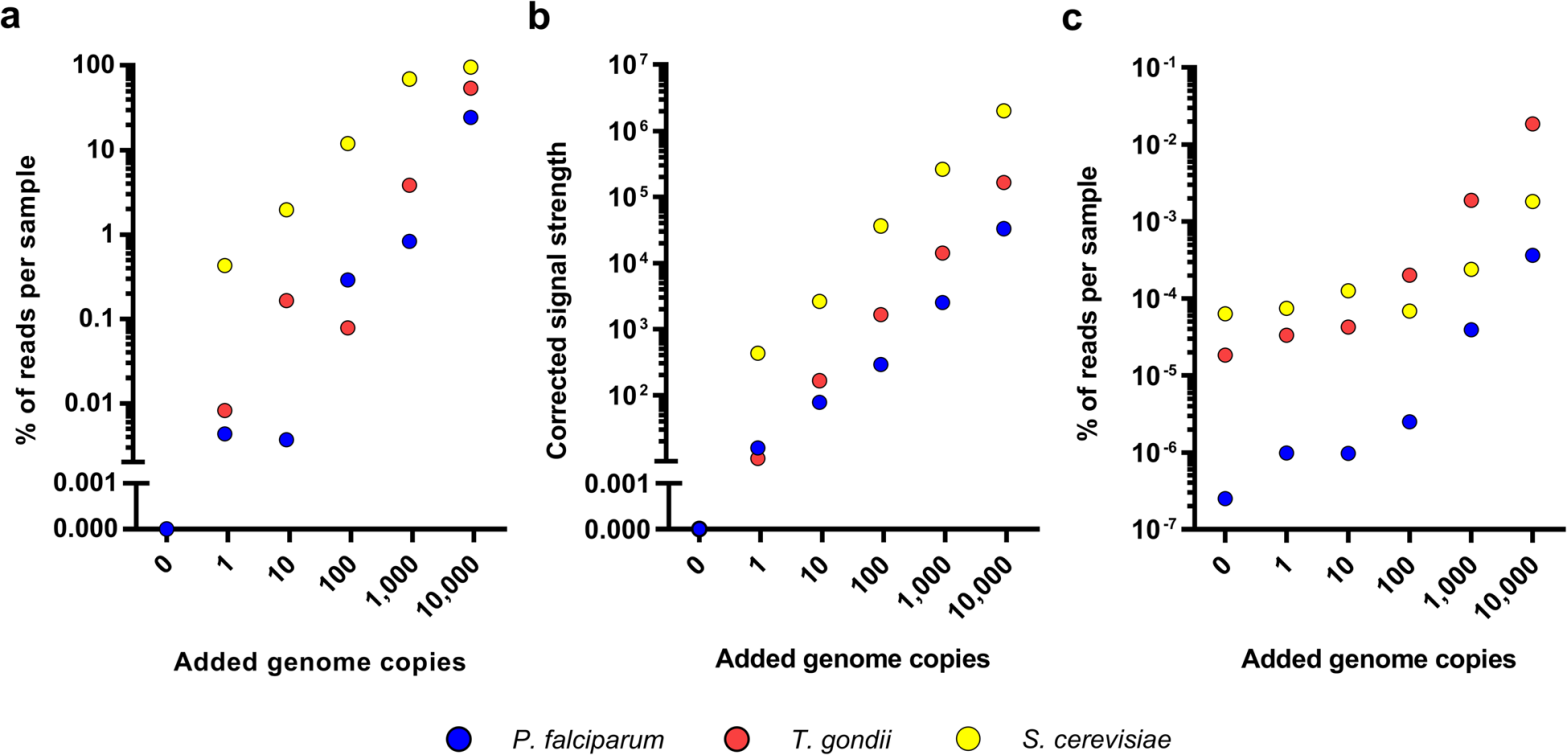
Sensitivity of 18S rRNA gene sequencing and HiSeq X Ten sequencing. Genome-copy detection limit determination of *P*. *falciparum*, *T*. *gondii*, and *S*. *cerevisiae* in positive control samples using a dual-index sequencing approach. **a** Representation of the percentage of 18S reads per eukaryotic positive control signal as measured. **b** Normalized representation of the absolute comparative strength of each eukaryotic signal after accounting for a different total eukaryotic signal strength in each of the positive control samples using 18S amplification. A value of 100,000 is representative of the human signal strength in each sample; *P*. *falciparum*, *T*. *gondii*, and *S*. *cerevisiae* signal strength values were calculated based upon the total remaining percentage of human reads in each positive control. **c** HiSeq X Ten genome-copy detection limit determination of *P*. *falciparum*, *T*. *gondii*, and *S*. *cerevisiae*

We then assessed the sensitivity of the optimized dual-index method by analyzing the signals from the positive controls to which 1, 10, 100, 1000, or 10,000 genome copies of *P*. *falciparum*, *T*. *gondii*, and *S*. *cerevisiae* had been added (Fig. 3). After normalizing for the total eukaryotic signal strength per positive control, this experiment demonstrated a near-perfect linear correlation between the number of genome copies added and the signal obtained for all three species (Fig. 3b). Moreover, with our approach, this correlation held to a single genome copy for all three eukaryotic species. For comparison, we also sequenced the same positive control samples using the Illumina HiSeq X Ten, aiming for 30-fold coverage of the human genome from the placenta. Each sample was run on a single lane on the HiSeq X Ten, resulting in approximately 200 million reads per sample. The detection limit for *P*. *falciparum* and *T*. *gondii* was ~ 100 added genome copies (Fig. 3c, represented by 10 and 827 reads respectively) and was higher for *S*. *cerevisiae* at more than 1000 genome copies.

### Placental infection and pregnancy complications

**Fig. 4:**
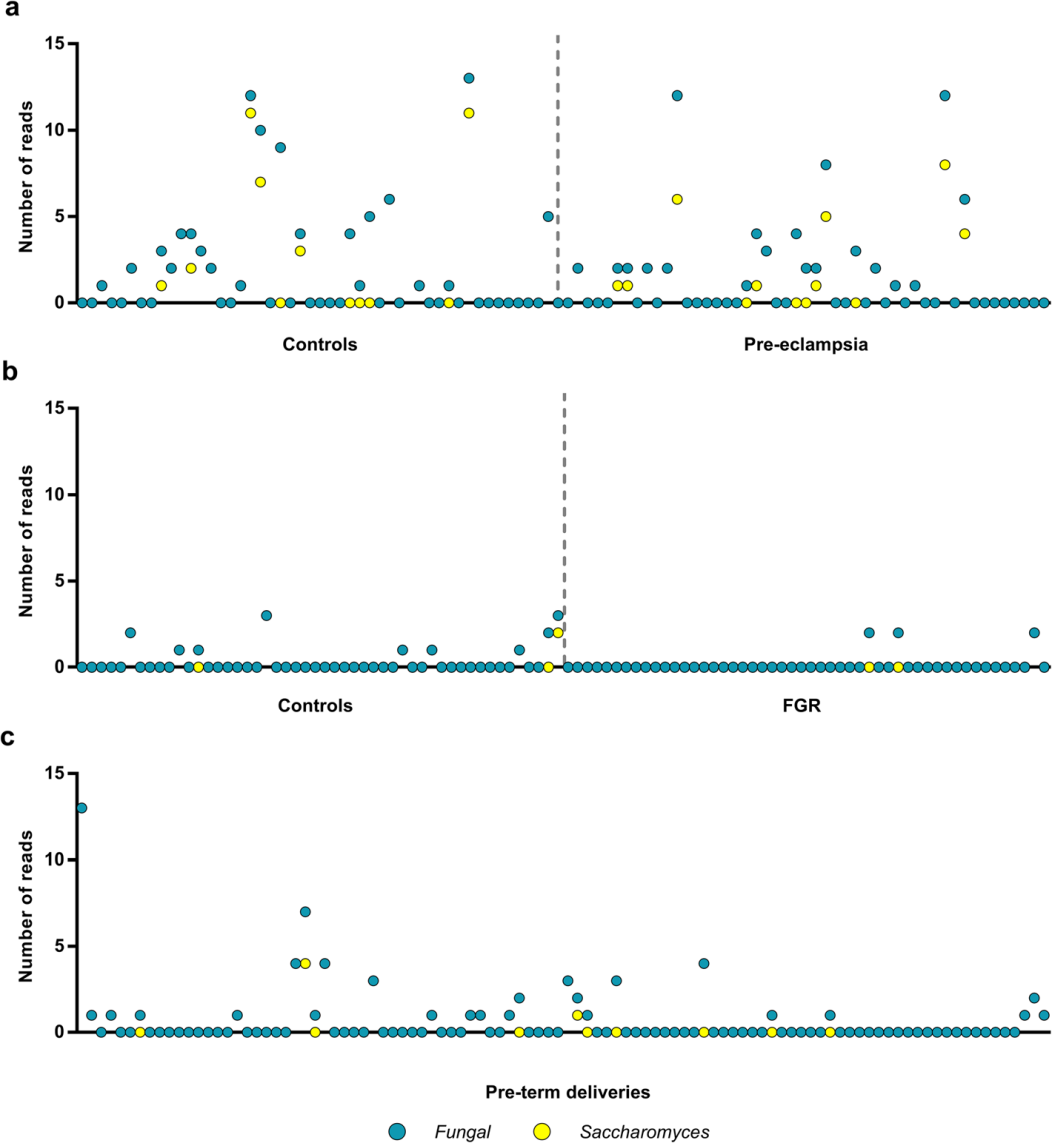
Detection of fungal reads in placental tissue in pregnancy complications with dual-index primers. **a** Pre-eclampsia and matched controls (*n* = 98), **b** fetal growth restriction (FGR) and matched controls (*n* = 100), and **c** pre-term deliveries (*n* = 100). The *Y*-axis represents the number of reads assigned to being of possible fungal origin (turquoise) and which were assigned to the *Saccharomyces* genus (yellow if different from the total number of fungal reads). None of the signals detected reached the 0.1% detection limit or a minimum of 100 fungal reads. The DNA isolations were performed using a single lot of DNA isolation kits but four different boxes. Matched cases and controls were processed in parallel on the same day, using the same box of laboratory reagents, and sequenced together in the same pool of 18S rRNA amplicons. Most pre-eclampsia matched cases and controls were sequenced together in one pool; therefore, the higher number of fungal reads detected may be related to differences in sequencing runs

Having optimized a sensitive method for the detection of 18S rRNA genes from eukaryotic microbes in human tissue, we then analyzed placental samples from (i) 50 cases of FGR and 50 matched controls, (ii) 49 cases of severe pre-eclampsia and 49 matched controls, and (iii) 100 cases of preterm delivery. Using the MiSeq dual-index 18S rRNA gene detection approach, none of the 298 samples demonstrated read counts indicative of even one genome copy for a potential eukaryotic pathogen. We estimated the upper bounds of the ability of 0/50 and 0/100 to rule out an association between eukaryotic infection and the respective outcomes using the binomial confidence interval. These were 0.0 to 7.1% and 0.0 to 3.6% of cases, respectively. *Saccharomyces* reads were the most commonly detected non-human eukaryotic reads. However, no sample had more than 15 *Saccharomyces* reads (Fig. 4), compared with the 163 reads observed when a control sample was spiked with a single genome copy of *Saccharomyces*.

## Discussion

In conducting this study, we identified a number of important lessons for other researchers searching for evidence of eukaryotic infections. First, the detection of non-human signal needs to be optimized for extremely low signals. Second, we found that 18S rRNA gene amplification is much more sensitive (and cost-effective) than deep sequencing. Third, we found that it is critical to employ unique indexes on the forward and reverse primers. The initial unexpected detection of *P*. *falciparum* and *T*. *gondii* signals in healthy placental samples turned out to be a sequencing artifact that was solved by switching from a single-index to a dual-index sequencing approach. Hence, the EMP-based, single-indexing approach is not suitable for pathogen identification in human samples as the presence of a single strongly positive sample in an 18S sequencing run will result in false positivity of this same signal in multiple other samples. However, our modified protocol allows detection of DNA from eukaryotic microbes present in very low amounts in multiple human biopsies multiplexed in a single sequencing lane. The false-positive *P*. *falciparum* and *T*. *gondii* signals were likely due to barcode cross-contamination of the primers. This is in line with previous reports [22], further highlighting the importance of dual-index sequencing approach.

Common approaches for the diagnosis of fungal diseases are PCR-based amplification, either pan-fungal or species specific, or antibody-based detection [23]. The detection limit of PCR-based techniques is generally between 5 and 10 genomic copies for a similar amount of tissue [24], but a single genome copy sensitivity can be obtained using electrochemically labeled DNA probes or with immunoassays [25, 26]. The loop-mediated isothermal amplification technique (LAMP) has similarly achieved a single genome copy sensitivity of *Toxoplasma gondii* which is an improvement over a PCR-based assay [27]. *Plasmodium* qPCR detecting high-copy telomere-associated repetitive elements has also achieved single genome copy sensitivity [28]. Each of these methods utilizes genus-specific primers whereas our modified protocol is generally applicable and does not require any prior knowledge. It is able to detect these various eukaryotes at a sensitivity of 1 genomic copy in multiple human biopsies (dominated by human DNA) in a single sequencing lane.

Regarding the *Saccharomyces* reads (and other minor signals detected in these 18S runs), further consideration indicates that these were likely to be the result of reagent contamination by eukaryotic DNA of environmental origin. It is well recognized that bacterial DNA of environmental origin is a common contaminant of laboratory kits. Amplification of these bacterial contaminants is a hazard in 16S or metagenomics analyses, especially in samples of low (microbial) biomass [29, 30]. Given that the saprophytic *Saccharomyces* genus is ubiquitous in nature and because *Saccharomyces* reads were also found in the negative controls, we hypothesized that *Saccharomyces* DNA was present in low amounts in the laboratory reagents employed [7]. A single *Saccharomyces cerevisiae* genome contains 100–200 copies of the 18S rRNA gene [31]. This probably explains why we were able to detect a single genome copy with our improved 18S rRNA gene MiSeq method, more readily than the other positive control species. Detection of fungal signals well below the 100 reads threshold with this method suggests that the sequenced DNA is derived from low-level environmental contamination rather than a true placental signal. The artificial nature of the signal is supported by the batch effect observed when comparing these putative contaminant signals in DNA extractions using different reagent boxes (Fig. 4). Consistent with this interpretation, the sample with the highest number of *Saccharomyces* reads (69) was a negative control. Therefore, the data do not support the hypothesis that placental eukaryotic infection is involved in the etiology of placental dysfunction or that there is any form of eukaryotic microbiota in the healthy placenta. However, the findings do indicate that 18S rRNA analysis is also prone to detecting contaminants in laboratory kits.

The positive control experiments show a need for having an internal positive control signal within each sample and for subsequent total signal strength normalization. In the case of the placental samples, signal strength normalization appears to be largely a moot point, but in other types of samples (with real signals), it is important to ascertain the actual number of cells of a microorganism within a sample (tissue) and not the relative abundance as compared to other microorganisms for purposes of biological relevance. In the case of 18S rRNA gene studies of human tissue, the human signal provides a control signal to allow quantification. This allows one to compare the relative strengths of the other signals found within these samples. The actual number of microorganisms that are present in the sample can then be estimated by comparison with positive controls, i.e., non-infected samples spiked with a known number of genome copies of the given organism. The use of a positive control may be particularly important in non-tissue samples, as there is no equivalent to human 18S and the addition of a positive control to all samples would facilitate quantification of the relative eukaryotic signal strengths.

Sequencing of the 18S rRNA gene allows for identification of eukaryotic organisms, such as fungi and protozoa, present within a given sample. Variable region 9 (which has been amplified in this study using the EMP primers) has previously been shown to be one of the better regions to sequence for the identification of eukaryotic diversity [32]. However, other regions of the 18S rRNA gene or associated areas may also be utilized to identify microbial DNA. As example of such other approaches are primers directed towards internal transcribed spacer (ITS) region. Targeted sequencing of the ITS region has previously been shown suitable for identification of fungal species [33–35].

In this study, we further demonstrate that FGR, pre-eclampsia, and pre-term birth were not associated with placental eukaryotic infections. No non-human 18S rRNA signals were detected in any of the placental samples that even came close to the 18S rRNA stipulated 0.1% detection limit (~ 100 reads). The sample size allowed us to rule out this being a factor in more than 7% of cases of FGR or pre-eclampsia or 3.6% of cases of preterm birth. Hence, these data do not support the hypothesis that placental eukaryotic infection may be involved in the etiology of placental dysfunction in the samples studied. It remains a possibility that this could be a rare cause of such complications; that it could be a more common cause in other settings, or that a eukaryotic pathogen eluded detection by methods employed in this study.

## Conclusions

The critical importance of utilizing optimized protocols and a dual-index sequencing approach when analyzing samples with a low eukaryotic microbe biomass has been illustrated in this study. We also demonstrate that placental infections with eukaryotic microbes/pathogens are unlikely as a (common) underlying cause of the pregnancy complications FGR, pre-eclampsia, or pre-term birth.

## Additional file

Additional file 1: Supplemental Figures and Tables. (DOCX 457 kb)

## Abbreviations

EGA: European Genome-phenome Archive
EMP: Earth Microbiome Project
ENA: European Nucleotide Archive
FGR: Fetal growth restriction
ITS: Internal transcribed spacer
LAMP: Loop-mediated isothermal amplification technique
POPs: Pregnancy Outcome Prediction study
SEM: Standard error of mean
