# Correction to: Detecting eukaryotic microbiota with single-cell sensitivity in human tissue

**DOI:** 10.1186/s40168-018-0576-3

**Published:** 2018-10-20

**Authors:** Susanne Lager, Marcus C. de Goffau, Ulla Sovio, Sharon J. Peacock, Julian Parkhill, D. Stephen Charnock-Jones, Gordon C. S. Smith

**Affiliations:** 1 Department of Obstetrics and Gynaecology, University of Cambridge, National Institute for Health Research Cambridge Biomedical Research Centre, Cambridge, UK; 2 Centre for Trophoblast Research (CTR), Department of Physiology, Development and Neuroscience, University of Cambridge, Cambridge, UK; 3 Wellcome Trust Sanger Institute, Cambridge, UK; 4 Department of Medicine, University of Cambridge, Cambridge, UK; 5 London School of Hygiene and Tropical Medicine, London, UK

## Correction

The author reported an error in an equation in the article [1].

The incorrect formula shown is the following:

$$ Correction\ factor=\frac{1+\left(99.9- Human\left(\%\right)\right)}{Human\ \left(\%\right)} $$

The correct formula should be:

$$ Correction\ factor=1+\frac{99.9- Human\left(\%\right)}{Human\ \left(\%\right)} $$
