# Supplementary material for: Detecting eukaryotic microbiota with single-cell sensitivity in human tissue
Source: Microbiome. 2018 Sep 1;6:151. doi: 10.1186/s40168-018-0529-x (PMC6119588; doi:10.1186/s40168-018-0529-x)
Supplement: Supplementary file 1 — Supplemental Figures and Tables. (DOCX 457 kb) [file 40168_2018_529_MOESM1_ESM.docx]

***Figure S1***

***
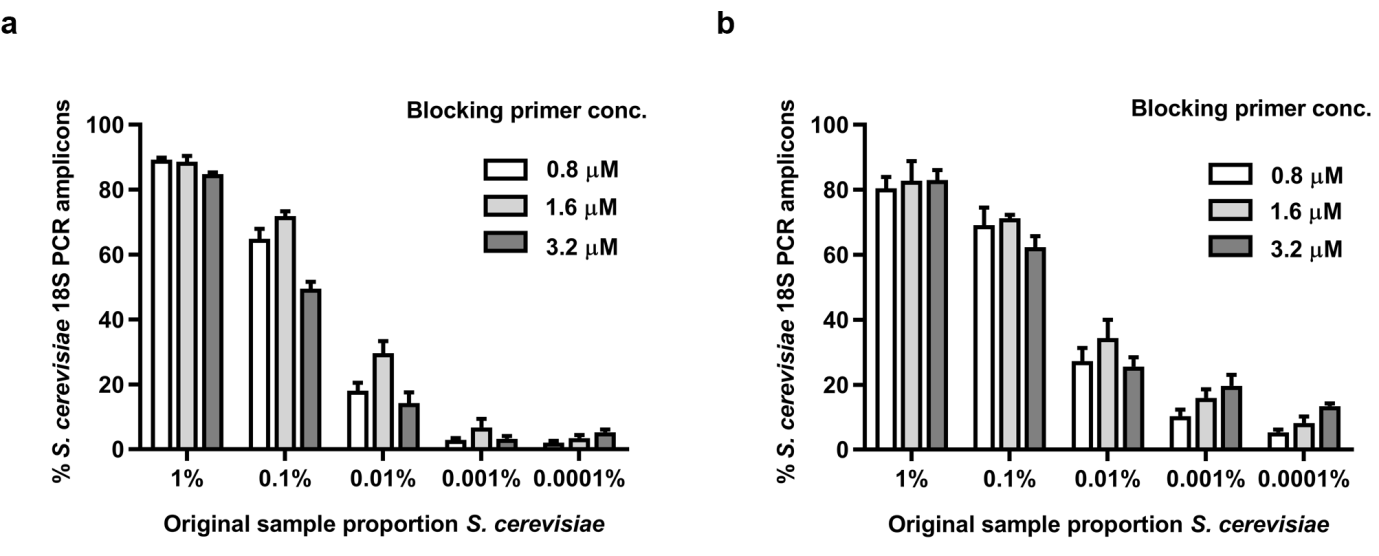
***

**Figure S1 Optimization of blocking primer annealing temperature and concentration.** The EMP protocol utilizes a mammal blocking primer annealing temperature of 65°C and blocking primer concentration of 1.6 µM. Different blocking primer concentrations were tested at two annealing temperatures for the 18S rRNA gene amplification. Graphs show proportion of *S. cerevisiae* 18S PCR fragments as determined by Q-PCR. (**a**) Blocking primer annealing temperature at 61°C. (**b**) Blocking primer annealing temperature at 65°C. Data presented as mean + SEM, n=4 (except 61°C, 0.01% *S. cerevisiae*, blocking primer amount 3.2 µM, n=3).

***Figure S2***

*
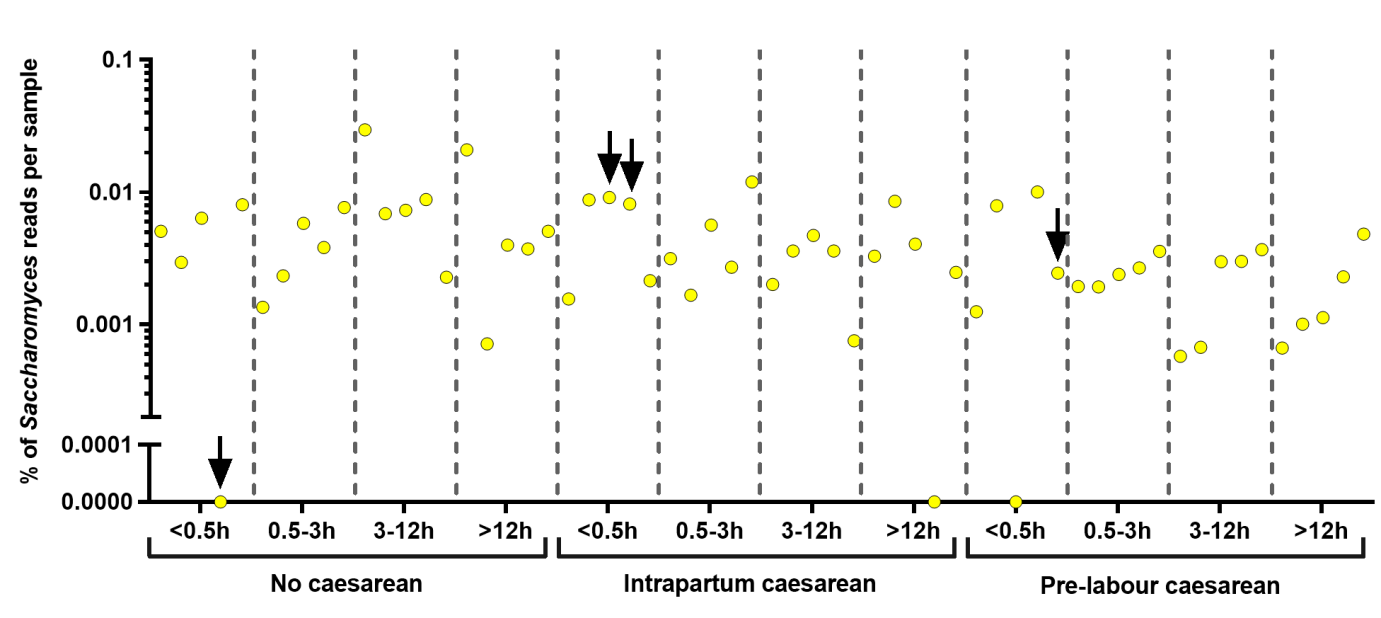
*

**Figure S2 Effect of mode of delivery and sample collection time on non-human 18S rRNA gene reads detected.** In order to determine the possible effects of mode of delivery and sample collection time, placental samples from healthy pregnancies were analyzed. Placental samples were collected <0.5 hour, 0.5-3 hours, 3-12 hours, or >12 hours after delivery. Mode of delivery as indicated. Human reads represented on average 99.88% of all reads. *Saccharomyces* reads were detected at low quantities (<0.1%). There was no association between mode of delivery or sample collection time and the number of reads of *Saccharomyces* detected. Sequencing was performed with single-index barcoded primers, n=60. The four placentas studied in more detail in figure 2 are indicated with arrows.

***Figure S3***


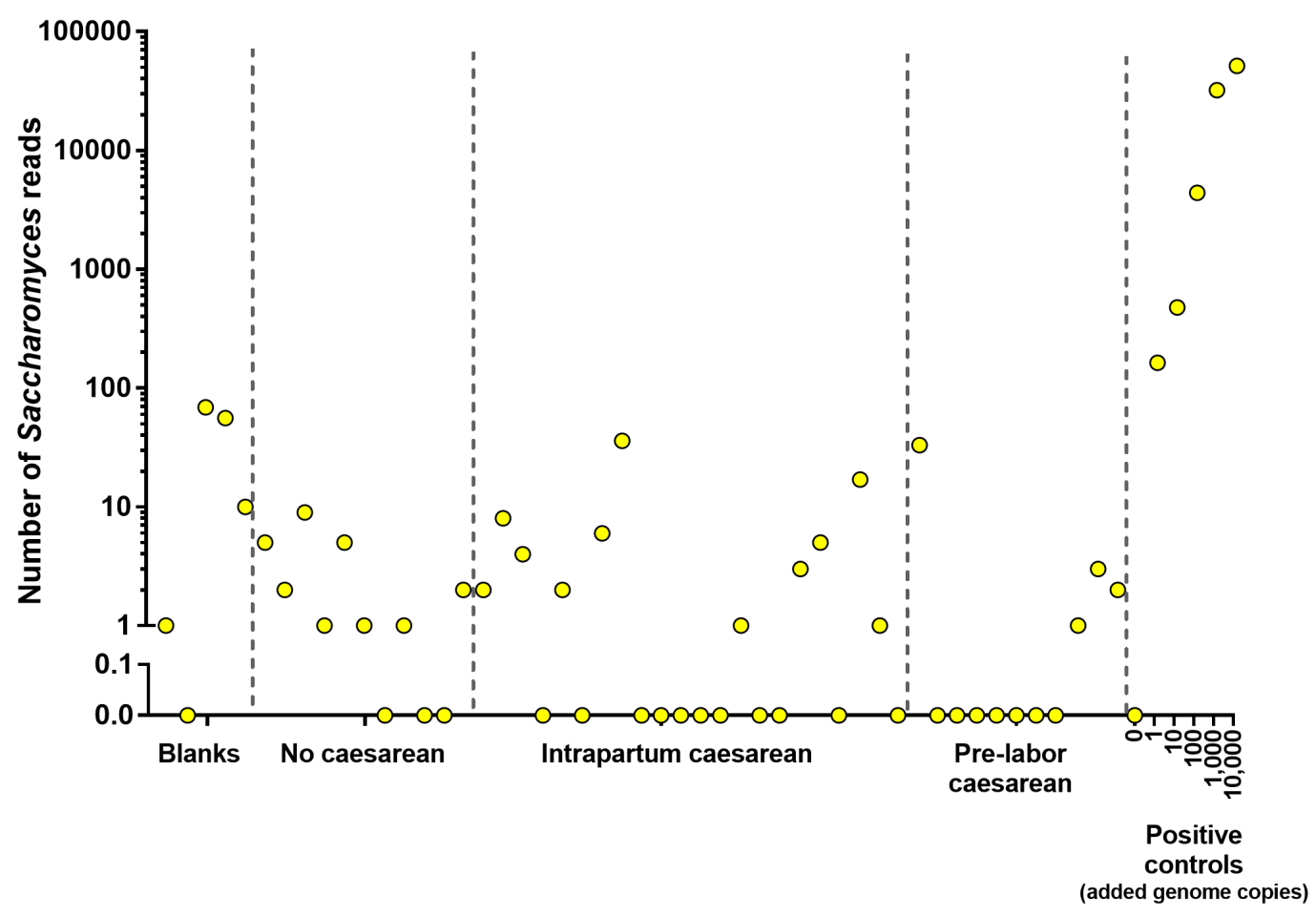


**Figure S3 Absolute number of *Saccharomyces* 18S rRNA gene reads.** Graph illustrate negative controls (blanks) (left; n=5), healthy placental samples (middle; n=44 placental biopsies from 4 women), and positive controls (right [0 to 10,000 added genome copies ordered from left to right]; n=6). With a dual-index sequencing signal remains in the positive control samples (right side of graphs; n=5), in placental samples as well as negative controls (blanks). The number of 18S rRNA gene reads in the blanks and placental samples were all below the number of reads detected in the positive control containing one added genome copy of *S. cerevisiae*.

***Table S1***

|  | Pre-labour Caesarean | Intrapartum Caesarean | No Caesarean |
| --- | --- | --- | --- |
| *N* | 20 | 20 | 20 |
| *Maternal BMI (kg/m^2^)* | 22 (21 to 25) | 26 (22 to 27) | 23 (21 to 25) |
| *Maternal age (years)* | 31 (28 to 36) | 31 (28 to 35) | 29 (27 to 31) |
| *Gestational age (weeks)* | 39 (39 to 40) | 41 (40 to 42) | 41 (40 to 41) |
| *Fetal sex: female* | 12 (60%) | 9 (45%) | 9 (45%) |
| *Smoking* | 2 (10%) | 2 (10%) | 2 (10%) |
| *Placenta collection time*  <0.5 hour  0.5-3 hours  3-12 hours  >12 hours | 5 (25%)  5 (25%)  5 (25%)  5 (25%) | 5 (25%)  5 (25%)  5 (25%)  5 (25%) | 5 (25%)  5 (25%)  5 (25%)  5 (25%) |
| *Age stopped FTE (years)* | 20.5  (17.0 to 23.0) | 22.0  (18.0 to 27.0) | 21.0  (18.0 to 23.0) |
| *Missing* | 0 (0%) | 1 (5%) | 2 (10%) |
| *Maternal height (cm)* | 164.0  (161.5 to 173.0) | 163.5  (163.0 to 169.5) | 167.5  (163.0 to 170.5) |
| *Deprivation quartile* |  |  |  |
| *1 (lowest)* | 7 (35%) | 3 (15%) | 2 (10%) |
| *2* | 1 (5%) | 4 (20%) | 9 (45%) |
| *3* | 8 (40%) | 6 (30%) | 4 (20%) |
| *4 (highest)* | 3 (15%) | 7 (35%) | 5 (25%) |
| *Missing* | 1 (5%) | 0 (0%) | 0 (0%) |
| *Ethnicity* |  |  |  |
| *Non-white* | 0 (0%) | 1 (5%) | 1 (5%) |
| *White* | 19 (95%) | 19 (95%) | 19 (95%) |
| *Missing* | 1 (5%) | 0 (0%) | 0 (0%) |
| *Married* | 12 (60%) | 12 (60%) | 15 (75%) |
| *Any alcohol consumption* | 2 (10%) | 0 (0%) | 0 (0%) |
| *Type I or type II diabetes* | 0 (0%) | 0 (0%) | 0 (0%) |
| *UtA Doppler mean PI*  *highest decile* | 1 (5%) | 2 (10%) | 3 (15%) |
| *Missing* | 2 (10%) | 1 (5%) | 0 (0%) |
| *Birth weight (g)* | 3343  (2998 to 3595) | 3660  (3510 to 3785) | 3600  (3398 to 3780) |

Median (interquartile range) or number (%) are given as appropriate. Smoking, maternal age and BMI were recorded at the booking appointment (~12 weeks of gestation) and other maternal characteristics were obtained from the 20 week questionnaire. BMI = body mass index, FTE = full-time education, UtA = uterine artery, PI = pulsatility index.

### ***Table S2***

|  | **FGR** | **Control** | **FGR vs. Control** | **PE** | **Control** | **PE vs. Control** | **Pre-term** |
| --- | --- | --- | --- | --- | --- | --- | --- |
| *N* | 50 | 50 |  | 49 | 49 |  | 100 |
| *Maternal BMI (kg/m^2^)* | 24.8  (22.8 to 28.8) | 24.7  (22.4 to 26.7) | 0.89  (-1.95 to 3.71) | 24.2  (22.7 to 27.5) | 23.8  (22.8 to 26.7) | 0.31  (-0.84 to 1.88) | 25.0  (22.8 to 28.5) |
| *Maternal age (years)* | 30.2  (26.7 to 33.7) | 30.7  (27.6 to 32.6) | -0.24  (-2.51 to 2.41) | 30.1  (26.2 to 33.8) | 29.9  (26.7 to 33.2) | -0.21  (-1.65 to 2.34) | 30.8  (27.0 to 34.1) |
| *Gestational age (weeks)* | 40.2  (39.1 to 41.1) | 40.2  (39.4 to 41.0) | -0.14  (-0.29 to 0.14) | 39.9  (38.7 to 41.0) | 40.0  (39.1 to 41.0) | -0.29  (-0.86 to 0.29) | 34.1  (31.9 to 35.6) |
| *Sample collection time (hours)* | 1.2  (0.2 to 3.7) | 0.7  (0.2 to 3.5) | 0.00  (-0.10 to 0.30) | 4.1  (0.5 to 9.0) | 3.8  (0.3 to 7.1) | 0.20  (-0.10 to 0.90) | 0.8  (0.2 to 9.1) |
| *Fetal sex: female* | 27 (54%) | 27 (54%) | 0 | 22 (45%) | 22 (45%) | 0 | 42 (42%) |
| *Smoking at booking (yes/no)* | 13 (26%) | 11 (22%) | 2 | 6 (12%) | 6 (12%) | 0 | 15 (15%) |
| *Age stopped FTE (years)* | 19.5  (17.0 to 23.0) | 21.0  (18.0 to 23.0) | -1.0  (-4.5 to 3.0) | 21.0  (18.0 to 23.0) | 21.0  (18.0 to 23.0) | -0.0  (-3.0 to 3.0) | 21.0  (18.0 to 23.0) |
| *Missing* | 0 (0%) | 2 (4%) |  |  |  |  | 5 (5%) |
| *Maternal height (cm)* | 165  (161 to 170) | 165  (161 to 169) | 0  (-7 to 5) | 165  (161 to 169) | 164  (159 to 168) | 1  (-6 to 6) | 164  (160 to 167) |
| *Deprivation quartile* |  |  | 32 |  |  | 33 |  |
| *1 (lowest)* | 15 (30%) | 10 (20%) |  | 17 (35%) | 11 (22%) |  | 25 (25%) |
| *2* | 7 (14%) | 10 (20%) |  | 10 (20%) | 10 (20%) |  | 22 (22%) |
| *3* | 11 (22%) | 14 (28%) |  | 11 (22%) | 13 (27%) |  | 31 (31%) |
| *4 (highest)* | 13 (26%) | 13 (26%) |  | 10 (20%) | 11 (22%) |  | 19 (19%) |
| *Missing* | 4 (8%) | 3 (6%) |  | 1 (2%) | 4 (8%) |  | 3 (3%) |
| *Ethnicity* |  |  | 9 |  |  | 4 |  |
| *Non-white* | 2 (4%) | 7 (14%) |  | 2 (4%) | 2 (4%) |  | 7 (7%) |
| *White* | 46 (92%) | 43 (86%) |  | 46 (94%) | 44 (90%) |  | 91 (91%) |
| *Missing* | 2 (4%) | 0 (0%) |  | 1 (2%) | 3 (6%) |  | 2 (2%) |
| *Married* | 31 (62%) | 35 (70%) | 22 | 35 (71%) | 37 (76%) | 20 | 66 (66%) |
| *Any alcohol consumption* | 1 (2%) | 2 (4%) | 3 | 0 (0%) | 0 (0%) | 0 | 4 (4%) |
| *Type I or type II diabetes* | 0 (0%) | 0 (0%) | 0 | 0 (0%) | 0 (0%) | 0 | 2 (2%) |
| *UtA Doppler mean PI*  *highest decile* | 12 (24%) | 5 (10%) | 14 | 12 (24%) | 4 (8%) | 16 | 22 (22%) |
| *Missing* | 2 (4%) | 1 (2%) |  | 0 (0%) | 1 (2%) |  | 7 (7%) |
| *Birth weight (g)* | 2610  (2375 to 2780) | 3505  (3340 to 3750) | -890  (-1092 to -760) | 3485  (3110 to 3770) | 3515  (3360 to 3690) | -155  (-345 to 220) | 2110  (1660 to 2525) |
| *Induction of labour* | 18 (36%) | 11 (22%) | 19 | 32 (65%) | 13 (27%) | 29 | 5 (5%) |
| *Mode of delivery* |  |  | 0 |  |  | 0 |  |
| *Vaginal* | 40 (80%) | 40 (80%) |  | 28 (57%) | 28 (57%) |  | 56 (56%) |
| *Intrapartum Caesarean* | 7 (14%) | 7 (14%) |  | 16 (33%) | 16 (33%) |  | 5 (5%) |
| *Prelabour Caesarean* | 3 (6%) | 3 (6%) |  | 5 (10%) | 5 (10%) |  | 39 (39%) |

Median (interquartile range) or number (%) are given as appropriate. FGR vs. Control and PE vs. Control: Median of differences (IQR) for continuous variables and Number of discordant pairs (among pairs with non-missing data) for binary variables. Smoking, maternal age and BMI were recorded at the booking appointment (~12 weeks of gestation) and other maternal characteristics were obtained from the 20 week questionnaire. One woman included in the SGA group also has PE. FGR = fetal growth restriction using customized reference centile, PE = preeclampsia using the ACOG 2013 definition, BMI = body mass index, FTE = full-time education, UtA = uterine artery, PI = pulsatility index.

***Table S3***

| GCA identifier | Species | Average no. of reads (SD) |
| --- | --- | --- |
|  | ***Total unmapped reads*** | 4,400,952 (567,036) |
|  | ***Unclassified unmapped reads*** | 4,289,086 (559,843) |
|  |  |  |
|  | ***Animalia*** |  |
| GCA_000258655.1 | *Pan paniscus* | 5,713 (240) |
| GCA_000004095.1 | *Hydra vulgaris* | 3 (2) |
| GCA_000003605.1 | *Saccoglossus kowalevskii* | 0.3 (0.5) |
| GCA_000002195.1 | *Apis mellifera* | 3 (2) |
| GCA_000002235.3 | *Strongylocentrotus purpuratus* | 11 (3) |
| GCA_000224145.2 | *Ciona intestinalis* | 0 |
| GCA_000146605.3 | *Meleagris gallopavo* | 13 (5) |
| GCA_000002035.3 | *Danio rerio* | 65,262 (4,919) |
| GCA_000457365.1 | *Biomphalaria glabrata* | 159 (356)* |
| GCA_000189315.1 | *Sarcophilus harrisii* | 5,387 (1,152) |
| GCA_000523025.1 | *Cynoglossus semilaevis* | 12 (5) |
|  |  |  |
|  | ***Gastrointestinal animalia*** |  |
| GCA_000975215.1 | *Caenorhabditis elegans* | 0 |
| GCA_000237925.2 | *Schistosoma mansoni* | 7 (4) |
|  |  |  |
|  | ***Pubic animalia*** |  |
| GCA_000006295.1 | *Pediculus humanus corporis* | 14 (13) |
|  |  |  |
|  | ***Plants*** |  |
| GCA_000005005.5 | *Zea mays* | 21 (37)** |
| GCA_000309985.1 | *Brassica rapa* | 67 (20) |
| GCA_000004075.2 | *Cucumis sativus* | 0.7 (1.1) |
| GCA_000002775.2 | *Populus trichocarpa* | 0.7 (0.5) |
| GCA_000331145.1 | *Cicer arietinum* | 0.9 (0.7) |
|  |  |  |
|  | ***Fungi*** |  |
| GCA_000002495.2 | *Magnaporthe oryzae* | 0.6 (0.6) |
| GCA_000002655.1 | *Aspergillus fumigatus* | 0 |
| GCA_000149445.2 | *Candida albicans* | 0.1 (0.4) |
| GCA_000226095.1 | *Thermothelomyces thermophila* | 0 |
| GCA_000149555.1 | *Fusarium verticillioides* | 0 |
| GCA_000146045.2 | *Saccharomyces cerevisiae* | 1,428 (2,653)*** |
|  |  |  |
|  | ***Excavata*** |  |
| GCA_000002825.1 | *Trichomonas vaginalis* | 0 |
| GCA_000499105.1 | *Naegleria fowleri* | 7 (3) |
| GCA_000498715.1 | *Giardia intestinalis* | 0 |
| GCA_000188675.2 | *Trypanosoma cruzi* | 0 |
| GCA_000002875.2 | *Leishmania infantum* | 0 |
| GCA_000002845.2 | *Leishmania braziliensis* | 0.1 (0.4) |
|  |  |  |
|  | ***Red algae*** |  |
| GCA_000350225.2 | *Chondrus crispus* | 0 |
| GCA_000397085.1 | *Porphyridium purpureum* | 0 |
|  |  |  |
|  | ***Amoebozoa*** |  |
| GCA_000413255.3 | *Physarum polycephalum* | 20 (8) |
| GCA_000826305.1 | *Acanthamoeba healyi* | 0 |
|  |  |  |
|  | ***Holozoa*** |  |
| GCA_000151315.2 | *Capsaspora owczarzaki* | 0.3 (0.5) |
| GCA_000188695.1 | *Salpingoeca rosetta* | 0.1 (0.4) |
|  |  |  |
|  | ***Apusozoa*** |  |
| GCA_000142905.1 | *Thecamonas trahens* | 2 (2) |
|  |  |  |
|  | ***Protists*** |  |
|  |  |  |
|  | ***Apicomplexan alveolate*** |  |
| GCA_000149715.2 | *Toxoplasma gondii* | 11,724 (27,222)*** |
| GCA_000002765.1 | *Plasmodium falciparum* | 236 (554)*** |
| GCA_000002415.2 | *Plasmodium vivax* | 8 (16)**** |
| GCA_000223845.4 | *Gregarina niphandrodes* | 0 |
| GCA_000497125.1 | *Spironucleus salmonicida* | 0 |
| GCA_000165395.1 | *Babesia bovis* | 0 |
|  |  |  |
|  | ***Oomycete*** |  |
| GCA_000142945.1 | *Phytophthora infestans* | 0 |
| GCA_000247585.2 | *Phytophthora parasitica* | 0 |
|  |  |  |
|  | ***Ciliated protozoan*** |  |
| GCA_000189635.1 | *Tetrahymena thermophila* | 0 |
|  |  |  |
|  | ***Marine centric diatom*** |  |
| GCA_000149405.2 | *Thalassiosira pseudonana* | 0 |
|  |  |  |
|  | ***Rhizaria*** |  |
| GCA_000512085.1 | *Reticulomyxa filosa* | 0 |
| GCA_000320545.1 | *Bigelowiella natans* | 2 (1) |

* The number of *Biomphalaria glabrata* reads is correlated directly with the *Toxoplasma gondii* spike concentration causing a large variation in the number of *Biomphalaria glabrata* reads. Slightly more than 1% of all *Toxoplasma gondii* reads end up assigned as *Biomphalaria glabrata* reads likely due to a contamination of the *Biomphalaria glabrata* genome with *Toxoplasma gondii* or perhaps due to an unknown similarity.

** Variation in the abundance of Zea mays detection is mainly due the presence of one relatively strong Zea Mays signal (103 reads) in one of the samples, which is also detected by 18S rRNA gene sequencing (62 reads). As suggested by others working on sequencing data of placental samples [pubmed: 27338728], we predict that such signals are possibly derived from pollen contamination.

*** Organism intentionally added as positive control

**** Phylogenetic similarity of *Plasmodium vivax* with *Plasmodium falciparum* causes a limited number of *Plasmodium falciparum* reads to be identified as *Plasmodium vivax*.
